# A multifaceted hand hygiene improvement program on the intensive care units of the National Referral Hospital of Indonesia in Jakarta

**DOI:** 10.1186/s13756-019-0540-4

**Published:** 2019-06-03

**Authors:** Yulia Rosa Saharman, Damiat Aoulad Fares, Souhaib El-Atmani, Rudyanto Sedono, Dita Aditianingsih, Anis Karuniawati, Joost van Rosmalen, Henri A. Verbrugh, Juliëtte A. Severin

**Affiliations:** 1 Department of Clinical Microbiology, Faculty of Medicine Universitas Indonesia / Dr. Cipto Mangunkusumo General Hospital, Jakarta, Indonesia; 2 Department of Medical Microbiology and Infectious Diseases, Erasmus MC University Medical Center Rotterdam, Dr. Molewaterplein 40, 3015 GD Rotterdam, The Netherlands; 3 Critical Care Division, Department of Anesthesia and Intensive Care, Faculty of Medicine Universitas Indonesia / Dr. Cipto Mangunkusumo General Hospital, Jakarta, Indonesia; 4 Department of Biostatistics, Erasmus MC University Medical Center Rotterdam, Rotterdam, The Netherlands

**Keywords:** Hand hygiene, Quality improvement, Guideline adherence, Intensive care unit, Indonesia

## Abstract

**Background:**

Hand hygiene (HH) is considered to be the single most effective measure in preventing healthcare-associated infections. However, HH compliance rates among nurses and doctors in hospitals are often very low. Few studies have addressed HH compliance in Indonesia, performed interventions to increase HH compliance, and none have had long-term follow-up. We, therefore, addressed this issue by performing long-term follow-up after a multifaceted intervention in the intensive care unit (ICU) setting.

**Methods:**

This was an observational, prospective, before-and-after intervention study (May–September 2014, February–April 2017). We measured HH knowledge and HH compliance before (at baseline) and directly after a multifaceted improvement program (post-intervention) and performed a re-evaluation three years later. The multifaceted improvement program included education, feedback, reminders, interviews and the use of role models. The study involved nurses and physicians working in two ICUs of the Dr. Cipto Mangunkusumo Hospital in Jakarta.

**Results:**

A total of 97 at baseline, and 72 at post-intervention HH knowledge questionnaires were completed. There was a statistically significant improvement in the median overall HH knowledge score at post-intervention (from 15 to 22, *p* < 0.001). There was no significant difference between the two ICUs. The overall HH compliance was 27% at baseline and significantly improved to 77% post-intervention (*p* < 0.001). For all five HH moments, the compliance of nurses and physicians separately improved significantly from the baseline phase to the post-intervention phase (*p* < 0.001), except for ‘moment 3’ (after body fluid exposure), for which baseline rates were already high. Most of the compliance rates were significantly lower in both groups of healthcare workers upon follow-up three years later. Overall, the HH compliance of the nurses was significantly better than the physicians’ compliance (*p* = 0.005).

**Conclusions:**

Our multifaceted improvement program, for nurses and physicians of the ICUs in the largest hospital of Indonesia, resulted in a significant improvement of the HH knowledge and HH compliance, but HH compliance levels waned over time after the intervention, indicating a need for continued monitoring and repeated interventions.

**Trial registration:**

The study was registered at www.trialregister.nl (No: 5541). Candidate number: 23527, NTR number: NTR5541, Date registered NTR: 22-DECEMBER-2015.

**Electronic supplementary material:**

The online version of this article (10.1186/s13756-019-0540-4) contains supplementary material, which is available to authorized users.

## Introduction

In both developed and resource-poor countries, healthcare-associated infections (HCAIs) have a large impact, especially those with multidrug-resistant (MDR) bacteria. [1, 2] One fourth of HCAIs involve patients in intensive care units (ICUs) and among them, the burden of infections by MDR microorganisms is the highest [3, 4].

The World Health Organization (WHO) identified the risk of acquiring a HCAI as being universal. The importance of hands in the transmission of infectious agents has been demonstrated. HH is considered to be the single most effective measure not only reducing the spread of microorganisms, but also preventing HCAIs [3]. Despite this fact, studies in developing countries, especially with a mid-level sociodemographic profile, have reported low compliance rates, including India: 3.0% [5], Vietnam: 43.6% [6], and Indonesia: city of Semarang (22.0 and 46.0%) [7], city of Malang (5.2, 10.1, and 24.1%) [8]. In a recent systematic review of the global HH literature, Erasmus et al. found a median HH compliance rate of 40% with a range of 4–100% [9].

A recent study on potential determinants of HH compliance showed that, besides the perception of the healthcare workers (HCWs) that there is a lack of evidence that HH is effective in preventing HCAIs, a lack of positive role models and social norms may hinder compliance [10].

Another study which took place on the island of Java, Indonesia, found that questionnaires in conjunction with site visits and interviews was a valuable strategy to identify trouble spots in the hospitals and to determine barriers of change that should be taken into account when planning interventions. [11]

The complexity of the process of behavioural change suggests that the application of multimodal, multifaceted strategies is necessary. Multifaceted strategies seem to result in a larger improvement of HH compliance (27–83%) as compared with using a single strategy (4–18%). [12] Education with written material, reminders, and continued feedback of performance can have an important effect on HH compliance. [12]

Using this information on HH improvement strategies, we developed a multifaceted improvement program to apply on the ICUs in the largest hospital of Indonesia. This multifaceted improvement program was based on WHO tools and included education, feedback, reminders, interviews and the use of role models [13]. The aim was to improve the HH knowledge and HH compliance of the nurses and physicians working on the ICUs of the Dr. Cipto Mangunkusumo Hospital in Jakarta. The study was part of a larger study focusing on the reduction of transmission of MDR bacteria in the ICU.

## Methods

### Study design

We performed an observational, prospective, before-and-after study in two ICUs of the largest hospital of Indonesia. Dr. Cipto Mangunkusumo Hospital is a 1200-bed university hospital located in Jakarta. At the time of this study up to 86% of patients did not have health insurance and had to pay for their hospital stay, medicines and laboratory tests. To improve HH knowledge and HH compliance among physicians and nurses we performed a multifaceted improvement program. This program was applied to the general ICU as well as to the ICU of the Emergency Department (ER-ICU), which is housed in a different part of the hospital but is managed by the same staff of Intensive Care Medicine.

The study consisted of four study phases (Table 1). In phase I, also referred to as baseline, which lasted from May 22nd – June 29th, 2014, we measured HH compliance by means of anonymous observations after which we performed HH knowledge tests through questionnaires completed between June 30th - July 7th, 2014. Phase II was the intervention that lasted from July 10th – August 29th, 2014, and which consisted of the implementation of a multifaceted improvement program that included education, feedback, reminders, interviews and the use of role models. Phase III was the post-intervention period from September 2nd – 30th, 2014, during which period HH knowledge was again measured through the same questionnaires as in phase I (from 1st-4th of September, 2014) and in which HH compliance was again measured through unobtrusive observations. Long-term evaluation of HH compliance was evaluated in phase IV (which lasted from February 20th – April 10th, 2017) by using the same protocols.

**Table 1:** Study phases and activities performed

Study phase	Activity	Date
Phase I: baseline	Hand hygiene compliance observations: healthcare workers	22 May- 29 June 2014
	Hand hygiene knowledge questionnaire: nurses and physicians	30 June-7 July 2014
Phase II: intervention	Multifaceted hand hygiene improvement program^a^	10 July – 29 August 2014
Phase III: post-intervention	Hand hygiene compliance observations: healthcare workers	2–30 September 2014
	Hand hygiene knowledge questionnaire: nurses and physicians	1–4 September 2014
Phase IV: long-term evaluation	Hand hygiene compliance observations: physicians and nurses	20 February – 10 April 2017

^a^Interventions are specified in Table 2

### Study setting and population

HCWs who participated in this study included all physicians on rounds in the ICUs, intensivists, all residents, nurses, students (medical students and nursing students), involved in patient care in these ICUs. HCWs were categorized into two categories, “physicians” and “nurses”, for the sake of simplicity. At the individual level, the mix of participants differed somewhat in each phase.

In the general ICU, 43 nurses and 8 physicians were employed. This ICU has fifteen beds. In the ER-ICU, 34 nurses, 6 student nurses and 6 physicians were employed. The ER-ICU has six beds. Two nurses and one physician were involved in the HH improvement strategies and were, therefore, not subjected to observations, interviews and questionnaires. At the start of the study, each patient bed had one wall-fixed, alcohol-based liquid hand disinfectant dispenser with alcohol-based hand rub based on the WHO formula [13]. Per two patient beds there was one sink with a medicated soap dispenser (Cutisoft^(@)^), containing 4% chlorhexidine, and one wall-fixed paper towel dispenser. There was no possibility of improving these basic facilities owing to the financial circumstances.

### Hand hygiene improvement strategies

Based on the study by M. Tromp et al., we developed an improvement program which included education, feedback and reminders [12]. Education was given in the form of interactive lessons developed by the WHO, including formal lectures, practical demonstrations and written material [13]. In each lesson, we emphasized that alcohol-based hand rub is superior to traditional handwashing as it requires less time, acts faster, irritates skin less often, and proved to be contributing significantly to sustainable improvement in compliance, which was associated with decreased infection rates [14]. Only when hands are visibly dirty or are visibly soiled with blood or other body fluids, hand washing with either non-antimicrobial soap and water or an antimicrobial soap and water was indicated [15].

Behavioural theories suggest that performance can by changed by feedback. In phase II and phase III personalized and non-personalized performance feedback was given to nurses and physicians of both wards. In several studies reminders were shown to have a sustainable effect on HH compliance. We gave reminders in the form of posters with handwashing messages placed on prominent sites in both ICUs [16].

In addition, we conducted interviews to determine the importance of social influence. The seven group interviews took 40–60 min. Each group interview consisted of 5–10 participants, both nurses and physicians. The interviews were led by a moderator. At the start of the interview, it was emphasized that there were no good or bad answers. All group interviews were recorded with a voice recorder. To ensure that all topics of interest were discussed an interview guide was developed and used in each interview.

All performed strategies are summarized in Table 2 and targeted the nurses and physicians of the general ICU as well as the ER-ICU.

**Table 2:** Hand hygiene improvement strategies used during the study

Improvement strategy	Date
Education	July 2014
Educational training	
Practical demonstration	
Written material	
Reminders	July 2014
Posters with hand hygiene reminders	
Interviews	August 2014
7 group interviews	
Performance feedback	July 2014–September 2014
Bar charts: hand hygiene compliance rates at baseline	
Individual feedback during observations	
Role models	September 2014
Assigned role models that instructed and stimulated their colleagues	

### Hand hygiene knowledge questionnaire

Based on the WHO’s ‘Hand Hygiene knowledge questionnaire’ a local questionnaire was developed (Additional file 1). The questionnaire was designed by the researchers and consisted of eight questions (including 17 sub-questions) about knowledge and five about attitude, perceived obstacles and self-reported behaviour. Two of the questions about perceived obstacles were developed by a Dutch clinical microbiologist. The questionnaire was translated into Indonesian after a pilot was tested by an Indonesian infection control nurse and physician. The questionnaire survey was carried out anonymously.

Participants completed the questionnaire during sessions at which the researchers were present to supervise. The ward was written on the form directly after a participant completed the questionnaire.

### Hand hygiene compliance observation

The gold standard to monitor compliance of HH is direct observation. [17]

There were four observers who worked in parallel in phase I, two observers had received training about the correct method of HH at the Unit Infection Prevention of the Erasmus MC University Medical Center that is accredited to do so. In phase I, two local fellows of clinical microbiology were enlisted as additional observers, who were trained and supervised by the principal investigator and the Erasmus MC trained observers. Prior to their scoring in the study these two fellows did a comparative trial run on observing compliance together with the trained observers from Erasmus MC and their results were compared using a kappa statistic to determine the interrater reliability. The two Erasmus MC trained observers scored HH compliance during phases I and III. The observers of the HH compliance in the phase IV, three years later, were local medical doctors recruited, trained and supervised in HH observations by one of the principal investigators (YRS).

Individual HCWs were observed during routine patient care by the observers with respect to potential HH opportunities available. The HCWs were not made aware that they were being observed. The nurses and physicians were unaware of the true reason for the presence of the observers at baseline, since we mentioned participation in a different study as a reason for having observers on the wards. In addition, to avoid a Hawthorne effect we elected, when designing the study, not to include the week in which questionnaires on hand hygiene were completed as part of the baseline observation period.

An observation list was developed based on the five moments for HH, and based on the WHO tools. The observations were carried out several times a week during differing time slots, but not during the weekend. Every episode of observation lasted about 30 to 60 min, but sometimes longer, also to reduce the Hawthorne effect.

The observation list contained 5 indications for HH: (1) before touching a patient, (2) before a clean/aseptic procedure, (3) after body fluid exposure risk, (4) after touching a patient (regardless of the use of sterile or nonsterile gloves), (5) after touching patient’s surroundings.

HH compliance was defined as hand disinfection using alcohol-based hand rub or washing hands with soap and water following one of the above-mentioned indications. The applied HH indication(s) and the performed HH action were marked on the observation list by the observers. [12] The outcomes of these observations were presented as percentages of compliance representing the fraction of the number of times when hand hygiene should have taken place correctly, and the number of times it had actually taken place correctly. The researchers, who were also the observers, recorded possible opportunities for HH over a period, which included observations at baseline (phase I), observations in the post-intervention phase (phase III), and long-term evaluation (observation by RK and M) (Phase IV), on both wards.

The targeted minimum number of observations for the baseline and post-intervention phases of the study was 1100 moments, as derived from a recent systematic review of previous HH compliance studies [9]. The WHO [13] points out that low numbers of observation are associated with wide ranges of the confidence intervals. When designing the study, we, therefore, elected to have at least 1100 observations in each of the pre- and post-intervention phases of the study to keep the confidence intervals around the observed, overall compliance rates narrow.

### Data analysis

For analysis of the questions regarding knowledge, correct answers were analysed as ‘correct’; incorrect answers, missing values (including ‘no answer’ and ‘do not know’) were all categorized as ‘incorrect’. The results of HH knowledge questionnaires were analysed in two ways: a) at the level of the individual HCW, i.e. number of correct answers per person, and b) at the level of individual questions asked, i.e. the percentage of correct answers given per question. For the first analysis each question was given the same value of one, such that each HCW received a score ranging from 0 to 25. To compare the scores of individual HCWs before and after the intervention (phase I and III, respectively), the scores were graphically plotted, and mean and median scores were calculated and compared using the Mann-Whitney test for independent samples. Because no data were stored on the identity of each HCW per phase, the scores in different phases could not be linked to each other, and a paired statistical analysis was thus not possible.

The performance of each of the 25 questions was analysed by scoring the percentage of participants providing the correct answer, thus these question-level scores ranged from 0 to 100%. The effect of the intervention on the percentage correct answers of each question was analysed using Fisher’s exact testing (two-tailed), and the Wilcoxon signed-rank test was used to compare the distribution of the percentage of correctly answered questions (per question) before and after the intervention.

For the questions about attitude, perceived obstacles and self-reported behaviour, the answers were used for the development of the improvement program. To determine the effects of the improvement strategy on compliance over time, we used linear mixed models for the repeated measurements of the compliance rates. The independent variables in this model were type of HCW (nurse or physician), phase (phase I, III, or IV), moment (a categorical variable for the 5 indications), and ward (general ICU or ER-ICU). All independent variables were coded as categorical variables. Two-way interaction effects between type of HCW, phase and moment were included in the model. Because the HCWs were not identified during data collection, the responses could not be linked between phases at the individual level. Instead we estimated the model for aggregated data, i.e. the outcome consisted of the average compliance rate (expressed as a percentage) for each combination of the variables type of HCW, phase, moment, and ward. This aggregation approach uses the fact that we can link outcomes over time at the level of a ward and allowed us to account for correlations between repeated measurements. The correlations were modelled by including a random intercept for each combination of type of HCW, ward, and moment in the model. We accounted for the substantial variation in the number of observations between different phases and moments by modelling the variance of the compliance rate as a function of the binomial variance (i.e. the observed compliance rate times (1-observed compliance rate) divided by the number of observations). The results of the linear mixed model analysis were summarized using the estimated marginal means, i.e. the predicted compliance rates adjusted for the effects of covariates, and presented graphically. These xestimated marginal means were compared between the phases for each combination of moment and type of HCW. The comparisons were adjusted for the effects of multiple testing using Tukey’s method.

Concordance between the observers was analysed using a kappa statistic. A kappa value of > 0.60 was considered good. For the linear mixed model, we used ‘R’, version 3.5.2 with packages nlme and emmeans. For other analyses, we used SPSS version 22.0 (SPSS, Inc., Chicago, IL.). In all analyses, a two-sided *p***-**value < 0.01 was considered statistically significant.

## Results

### Interviews

Prevention of cross-infections was named by the participants as the main advantage of HH. Dryness and soreness of hands after performing HH were the main disadvantages brought up by the participants.

A lack of social control with regard to HH compliance was mentioned by nurses as well as physicians. Nurses had no difficulties in approaching other HCWs, even physicians, about their HH behaviour. However, physicians reported to have difficulties in approaching for example senior staff members, because of ‘the culture’ in the hospital. In addition, they mentioned that noncompliance among physicians could arise from lack of strong evidence supporting the effectiveness of HH for prevention of HCAIs. Participants, nurses in particularly, mentioned the need for positive role models. The presence of negative role models, nurses or physicians noncompliant with HH, was reported as reason for their own noncompliance.

Furthermore, participants noted that strategically placed reminders (posters, labels with messages) would help increase and maintain compliance with HH. Also, participants advised to regularly change the reminders into different ones in order to be sure that the reminders remain effective.

### Hand hygiene knowledge

**Fig. 1:**
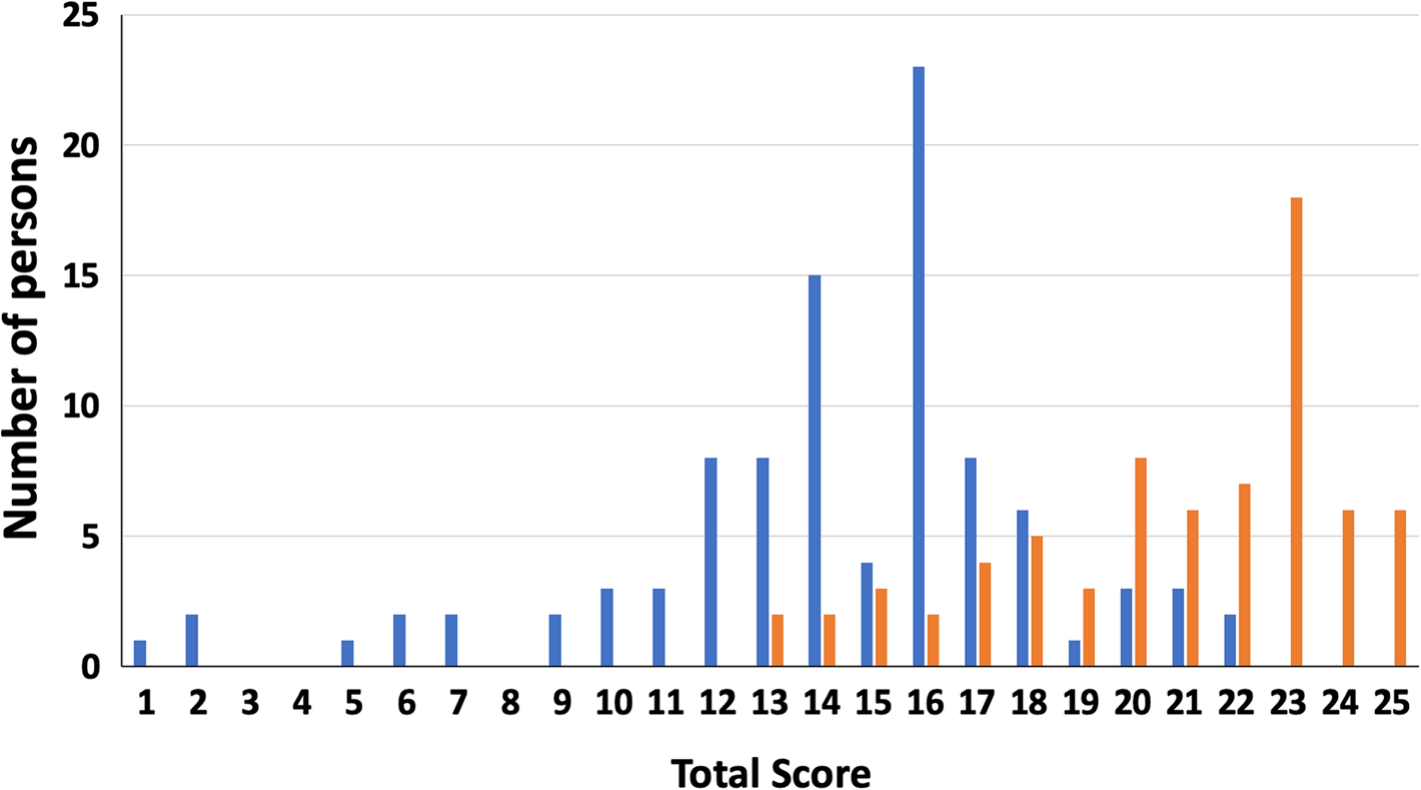
Effect of an educational intervention on hand hygiene knowledge of healthcare workers. Bars indicate the number of persons achieving indicated overall score derived from questionnaires taken during the baseline (blue bar) and post-intervention (orange bar) phases of the study

At baseline we measured the HH knowledge of 43 nurses and 8 physicians of the general ICU. At the ER-ICU 34 nurses, 6 physicians and 6 nurse students participated. The overall score of correctly answered HH knowledge questions for all HCWs and both wards combined ranged from 1 to 22, with a mean overall score of 14.3 (median 15; interquartile range (IQR) 13–16). Post-intervention we measured the HH knowledge of 32 nurses and 5 physicians of the general ICU. At the ER-ICU these were 31 nurses and 4 physicians. Of this group, the mean overall score of correctly answered knowledge questions for all HCWs and both wards combined was 20.8 (median 22; IQR 18–23), with a range from 13 to 25 (Fig. 1). Compared with baseline, there was a significant improvement in the overall mean HH knowledge score post-intervention (*p* < 0.001). There was no significant difference observed in the overall mean score between the general ICU and the ER-ICU (*p* = 0.692 by Mann-Whitney test, data not shown).

The multifaceted intervention resulted in significant improvement in HCW knowledge (median percent correct answer across all questions pre-intervention, 71% versus post-intervention phase, 89%; *p* < 0.001). Additional file 2: Table S1 displays the results for the 25 individual HH knowledge questions separately, at baseline as well as post-intervention. For 15 questions, significantly more correct answers were given in the post-intervention phase compared to baseline. Interestingly, the question (5c) with the 2nd lowest number of correct answers in the pre-intervention phase (14.4%) significantly improved its score but had the lowest score in the post-intervention phase (52.8%); this question addressed the effectiveness of hand rubbing compared to handwashing. Thus, even after education and hand-written evidence almost half of the respondents remained convinced that handwashing with water and soap is more effective than hand rubbing with alcohol. In contrast, the rate of correct answers to question 2 improved from a very low 4.0 to 75.0%, this question dealt with the most prevalent source of pathogens causing nosocomial infections.

### Hand hygiene compliance

In the general ICU and the ER-ICU combined, a total of 7187 HH opportunities were observed (Table 3). The observations between researchers and the clinical microbiology fellows were concordant (kappa 0.72) when tested in a pilot run in the general ICU.

**Table 3:** Number of opportunities for hand hygiene observed at baseline, in the post-intervention phase, and at long-term follow-up

	Baseline	Post-intervention	Long-term evaluation	Total
Before touching a patient	386	218	705	1309
Before a clean/aseptic procedure	212	171	241	624
After body fluid exposure risk	108	59	267	434
After touching a patient	692	299	703	1694
After touching patient’s surroundings	920	642	1564	3126
Total	2318	1389	3480	7187

The most frequently observed indications for HH were ‘after touching patient’s surroundings’ 3126/7187 (44%) and ‘after touching a patient’ (24%) (Table 3).

**Fig. 2:**
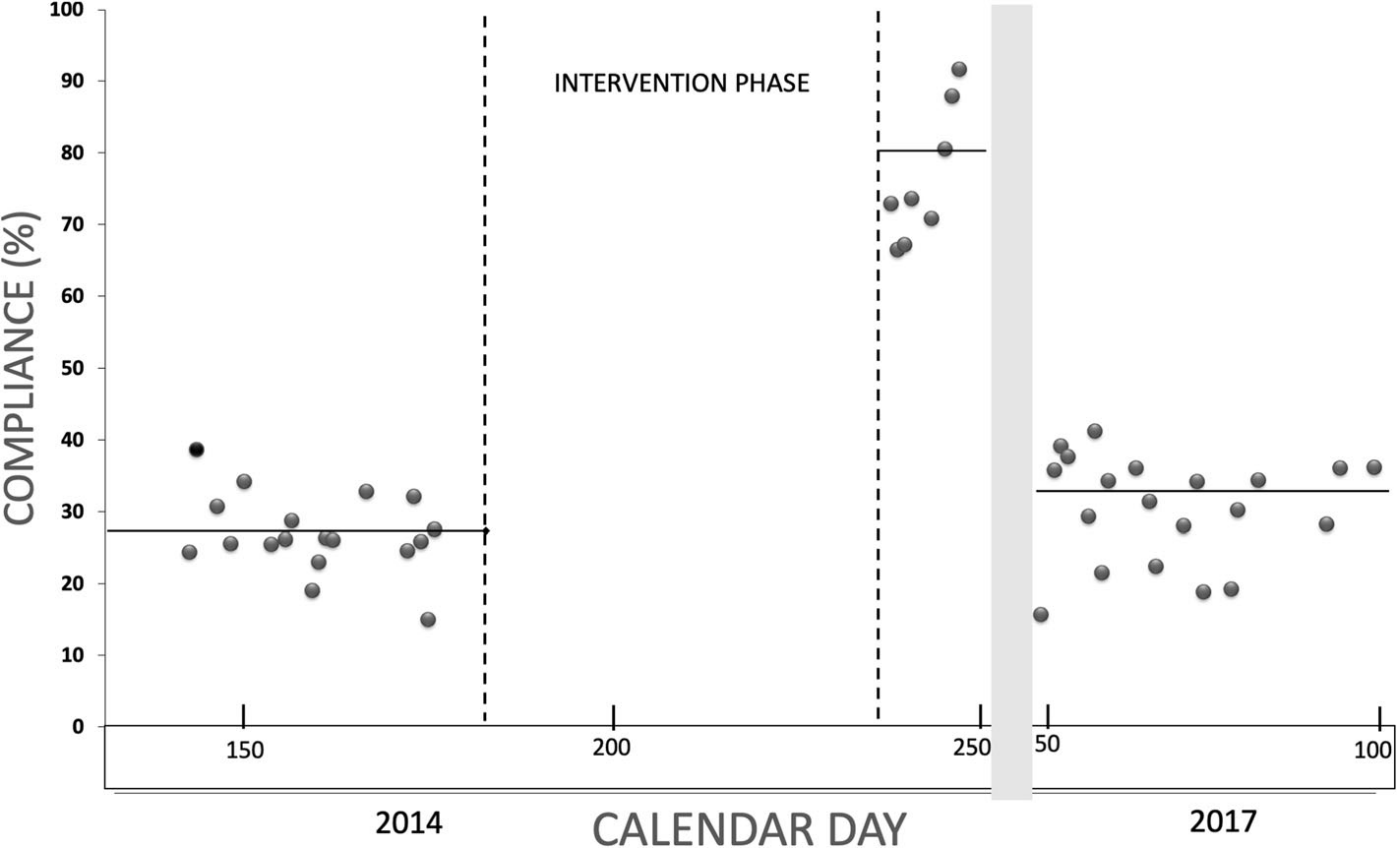
Time series of compliance rates per observation day. Dots indicate the compliance rates per observation day. The observations used to calculate the compliance rates per day were conducted in the baseline phase and the post-intervention phase in 2014, and in the long-term evaluation phase in 2017. The horizontal lines represent the average of compliance rate in each phase

**Fig. 3:**
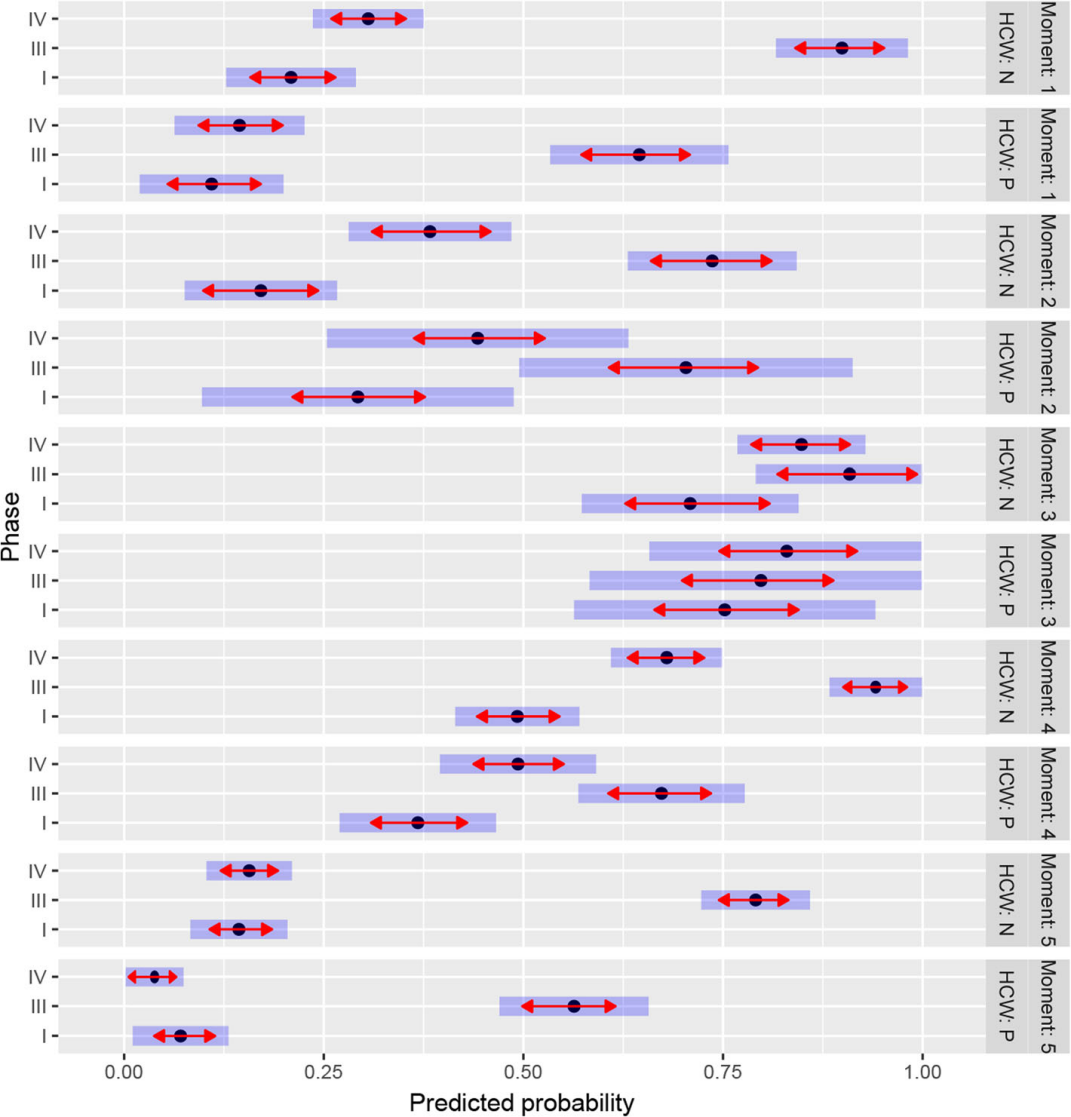
Predicted compliance rates by WHO moment of hand hygiene and by healthcare worker in the baseline phase (I) and post-intervention phases (III and IV). HCW, healthcare worker**;** HCW: N: nurse; HCW: P: physician. Moment 1 is before touching a patient. Moment 2 is before a clean/aseptic procedure. Moment 3 is after body fluid exposure risk. Moment 4 is after touching a patient. Moment 5 is after touching patient’s surroundings. Black dots indicate the estimated marginal means of compliance rate. The red arrows give information on the significance of the difference between phases. Overlapping red arrows within a block (i.e. a block of phase I, III, and IV) means a nonsignificant difference between phases, nonoverlapping arrows imply a significant difference. The arrows are adjusted for multiple testing using Tukey’s method. The blue bars are 95% confidence intervals, not adjusted for multiple testing

The number of HH opportunities at baseline, both wards combined, was 2318 with an overall compliance rate of 27%. In the post-intervention phase, the number of HH opportunities was 1389 with an overall compliance of 77%. Thus, the overall HH compliance improved significantly from baseline phase I to the post-intervention phase III, but had regressed to the baseline level again when evaluated at long-term follow-up (33% in phase IV, Fig. 2, Table 4). For all moments, the HH compliance of nurses and physicians separately improved significantly from phase I to phase III (*p* < 0.001), except for moment 3 (Fig. 3, Table 4). Over the whole observation period, however, the HH compliance rates were higher among the nurses than among the physicians (44% versus 27%, *p* = 0.005 in linear mixed model). There was no significant difference in compliance rate found between the two wards (*p* = 0.463, data not shown).

**Table 4:** Hand hygiene compliance rates by nurses and physicians observed for each of the 5 HH moments at baseline, post-intervention and at long-term evaluation

Moment, by healthcare worker	Compliance % (correct/total number)					
	Baseline	Post-intervention	Long-term evaluation	*p* (I-III)^a^	*p* (I-IV)^a^	*p* (III-IV)^a^
	(Phase I)	(Phase III)	(Phase IV)			
1. Before touching a patient						
Nurses	21 (58/276)	88 (115/131)	31 (174/569)	**< 0.001**	0.0657	**< 0.001**
Physicians	13 (14/110)	66 (57/87)	18 (24/136)	**< 0.001**	0.7147	**< 0.001**
2. Before a clean/aseptic procedure						
Nurses	17 (33/194)	75 (118/157)	37 (80/215)	**< 0.001**	**0.0032**	**< 0.001**
Physicians	39 (7/18)	50 (7/14)	54 (14/26)	**< 0.001**	0.0775	**0.0037**
3. After body fluid exposure risk						
Nurses	69 (64/93)	91 (49/54)	85 (204/239)	0.0383	0.1032	0.5811
Physicians	80 (12/15)	80 (4/5)	75 (21/28)	0.8608	0.5276	0.8958
4. After touching a patient						
Nurses	49 (257/529)	93 (181/194)	70 (390/560)	**< 0.001**	**< 0.001**	**< 0.001**
Physicians	39 (63/163)	72 (76/105)	43 (61/143)	**< 0.001**	0.0345	**0.0036**
5. After touching patient’s surroundings						
Nurses	15 (95/616)	80 (370/463)	15 (152/1043)	**< 0.001**	0.9007	**< 0.001**
Physicians	6 (18/304)	54 (96/179)	3 (18/521)	**< 0.001**	0.2311	**< 0.001**

^a^Based on the linear mixed model analysis. Significant changes in compliance are in bold character

We also analysed the sustainable effect of this intervention program by a long-term follow-up evaluation (phase IV). Most of the compliance rates were significantly lower in both groups of HCWs (phase III versus phase IV), except for moment 3 (after body fluid exposure risk), for which both physicians and nurses had high compliance rates at baseline and remained so in phase III and IV, albeit that these values were statistically not significantly higher.

## Discussion

This was an observational, prospective, before-and-after intervention study. The overall HH compliance rates at baseline were low in both ICUs (27%). The HH compliance level observed before intervention was similar to those reported from other countries that have a mid-level sociodemographic index [18, 5, 19, 20, 6], and other cities in Indonesia [7, 8].

Our study showed that overall a significant increase in the HH compliance of HCWs could be achieved using a multifaceted improvement program (compliance in post-intervention phase: 77%). In line with the report by Tromp et al. [12], we developed a multifaceted improvement program. Since this is a multifaceted approach, it is not possible to determine the contribution of each component to the observed improvement.

Duerink et al. introduced a multifaceted intervention study to improve compliance in a tertiary care hospital in the city of Semarang, Indonesia, and found this strategy increased HH compliance from 46.0 to 77.0% in the internal medicine ward and from 22.0 to 62.0% in the paediatric ward. [7] Santosaningsih et al. also performed interventions in a similar hospital in Malang, and improved HH compliance significantly in paediatric (24.0 to 44.0%) and internal medicine wards (5.0 to 19.0%), but also in the control ward, obstetrics-gynaecology, where no intervention had been performed (10.0 to 21.0%). [8] They concluded that role model training had the most impact. [8]

Our strategy was highly effective for the nurses as well as the physicians. Similar to previous studies [21], the overall level of HH compliance in our study was significantly higher among nurses (44%) than among physicians (27%). In the setting of our study, we noticed a clear difference in activities of nurses and physicians. Nurses play an important role in the daily care of the patients. Physicians have much fewer patient contacts. Therefore, the nurses have far more HH opportunities in their daily routine. This could explain why the nurses had a more pro-active attitude towards the improvement program with a higher HH compliance as result. This difference in HH compliance between nurses and physicians may also be due to the fact that not all physicians were convinced about the effectiveness of HH. Physicians mentioned that their noncompliance was associated with a perceived lack of evidence that hand hygiene is effective in the prevention of hospital-acquired infection, which could be an explanation for the inverse correlation found between the level of education and the rate of handwashing compliance. Better information about the available evidence might well promote better compliance. [16, 21]

In the analysis of the compliance rates per HH moment, there is a notable difference between ‘moment 3’ and the other four HH moments. The HH compliance rates for ‘moment 3’ at baseline (physicians: 80%, nurse: 69%), were already very high, compared to the HH compliance rates of the other HH moments. Moment 3 is defined as a HH indication after body fluid exposure risk. Other studies have shown that the HH behaviour of healthcare workers appears to be motivated by self-protection and a desire to clean oneself after a task that is perceived to be dirty, rather than protect their patients by proper HH before approaching the patients. This may explain the high HH compliance rate at baseline in our study. [21]

HH knowledge was indeed low at baseline, but showed a large increase in the post-intervention phase. Therefore, we hypothesize that education was an important component of the intervention.

Dryness and soreness of hands after performing HH were the main obstacles reported by participants of the interviews and in the HH knowledge questionnaire before the multifaceted intervention. The medicated soap and the hand alcohol suspensions did not contain moisturizers and emollients. Hand washing with 4% chlorhexidine medicated soap dries out the skin and results in pre-irritated skin. Applying alcohol-based hand rub to pre-irritated skin may cause a burning sensation. Because of the burning sensation the HCWs tend to reduce the use of alcohol-based hand rub and prefer hand washing with medicated soap, which is the underlying reason for the burning sensation. However, this fact was not stressed during the educational part of the intervention. Due to financial constraints, additional moisturizing hand creams were not available. Replacement of the medicated soap to plain mild soap would have been more in line with the WHO strategy.

The long-term effect of our multifaceted improvement program was assessed after three years. This follow-up showed that the intervention by and large did not have a sustainable effect on HH compliance, except ‘moment 3’ (after body fluid exposure risk) in both groups of HCWs. In Khatib et al. [22] the use of reminders showed a modest positive effect on HH compliance rates, but they were able to maintain higher compliance rates for a longer period of time. Other studies also showed that reminders have a modest sustaining effect on HH compliance rates. [16, 23] With the use of reminders and positive role models in our multifaceted improvement program, we strived to have a long-term effect on HH compliance rates.

Human behaviour is a complex process determined among others by knowledge about and attitude towards the behaviour, perceived social standards and self-efficacy. [11] Behavioural change is often viewed as a difficult process, also in the hospital. HCWs continue to fail in adherence to the guidelines for HH which hampers the reduction of HCAIs. [4] In several studies, the effectiveness of different HH improvement strategies have been described [17]. Erasmus et al. found that personal beliefs about the efficacy of HH and examples and norms provided by senior hospital staff are of major importance for HH compliance. They further reported that HH is most often performed after tasks that they perceive to be dirty, and personal protection appeared to be more important for compliance than patient safety. Physicians mentioned that their noncompliance arose from their belief that the evidence supporting the effectiveness of HH for prevention of HCAIs is not strong [21].

Some possible limitations of our study must be considered. For measuring the HH compliance, we used direct observations using the standard observation form as defined by the WHO. [3] Direct observations have limitations; they are time-consuming, manpower intensive and continuous monitoring is currently not feasible in this setting. The information provided probably represents a low percentage of all HH opportunities. By mentioning our participation to a different study as reason for the presence of the observers, the nurses and physicians were unaware of the true reason for the observations at baseline. Despite of our long periods of observations, observation bias and the Hawthorne effect cannot be fully excluded. Also, the effectiveness of HH on the prevention of HCAIs depends on HH technique in addition to HH compliance. [24] HH technique training was a part of our program, however, this was not evaluated. A full system change was not achieved by the intervention. State-of-the-art is to increase the use of alcohol-based hand rub and decrease hand washing. 4% Chlorhexidine medicated soap should have been removed from all wards. A mild liquid soap should have been provided instead of medicated soap. The observed decrease in HH compliance rates after the initial post-intervention phase could be, at least in part, the result of an incomplete system change.

## Conclusions

In conclusion, our multifaceted improvement program, for nurses and physicians of the ICUs in the largest hospital of Indonesia, resulted in a highly significant improvement in the HH knowledge and HH compliance, but maintaining high levels of HH compliance requires continuous monitoring and regular interventions.

## Additional files

Additional file 1: Hand hygiene knowledge questionnaire for healthcare workers (in English and Indonesian). (DOCX 243 kb)

Additional file 2: **Table S1.** Survey results regarding hand hygiene knowledge questions. (DOCX 17 kb)
