# Supplementary material for: A multifaceted hand hygiene improvement program on the intensive care units of the National Referral Hospital of Indonesia in Jakarta
Source: Antimicrob Resist Infect Control. 2019 Jun 3;8:93. doi: 10.1186/s13756-019-0540-4 (PMC6547605; doi:10.1186/s13756-019-0540-4)
Supplement: Supplementary file 1 — Hand hygiene knowledge questionnaire for healthcare workers (in English and Indonesian). (DOCX 243 kb) [file 13756_2019_540_MOESM1_ESM.docx]

You are in direct contact with patients on a daily basis and this is why we are interested in your **opinion** on health care-associated infections and hand hygiene.

- It should take you about 15 minutes to complete this questionnaire.
- Each question has **one answer only**.
- Please read the questions carefully and then respond spontaneously. Your answers are anonymous and will be kept confidential.
- **Short Glossary:**

**Alcohol-based handrub formulation:** an alcohol-containing preparation (liquid, gel or foam) designed for application to the hands to kill germs.
**Facility:** health-care setting where the survey is being carried out (e.g., hospital, ambulatory, long-term facility, etc).
**Handrubbing:** treatment of hands with an antiseptic handrub (alcohol-based formulation)
**Handwashing:** washing hands with plain or antimicrobial soap and water.
**Service:** a branch of a hospital staff that provides specified patient care.
**Ward:** a division, floor, or room of a hospital for a particular category or group of patients (it corresponds to the smallest segmentation of the health-care facility; one service can include multiple wards)**.**

**Date**:

**Gender**: □ Female □ Male

**Proffesion**: □ Nurse □ Nurse student □ Medical doctor □ Medical student □ Other

1. **Which of the following is the main route of cross-transmission of potentially harmful germs between patients in healthcare facility? (one answer only)**
2. □ **Healthcare workers’ hands when not clean**
3. □ Air circulating in the hospital
4. □ Patients’ exposure to colonized surfaces (i.e., beds, chairs, tables, floors)
5. □ Sharing non-invasive objects (i.e., stethoscopes, pressure cuffs, etc.) between patients
6. **What is the most frequent source of germs responsible for healthcare-associated infections? (one answer only)**
7. □ The hospital’s water system
8. □ The hospital air
9. **□ Germs already present on or within the patient**
10. □ The hospital environment (surfaces)
11. **Which of the following hand hygiene actions prevents transmission of germs to the patient?**
12. □ Before touching a patient □ Yes □ No
13. □ Immediately after risk of body fluid exposure □ Yes □ No
14. □ Immediately before a clean/aseptic procedure □ Yes □ No
15. □ After exposure to the immediate surroundings of a patient □ Yes □ No
16. **Which of the following hand hygiene actions prevents transmission to the healthcare worker?**
17. □ Before touching a patient □ Yes □ No
18. □ Immediately after risk of body fluid exposure □ Yes □ No
19. □ Immediately before a clean/aseptic procedure □ Yes □ No
20. □ After exposure to the immediate surroundings of a patient □ Yes □ No
21. **Which of the following statements on alcohol-based handrub and handwashing with soap and water are true?**
22. Handrubbing is more rapid for hand cleansing than handwashing □ True □ False
23. Handrubbing causes skin dryness more than handwashing □ True □ False
24. Handrubbing is more effective against germs than handwashing □ True □ False
25. Handwashing and handrubbing are recommended to be performed in sequence □ True □ False
26. **What is the minimal time needed for alcohol-based handrub to kill most germs on your hands?**
27. □ 20 seconds
28. □ 3 seconds
29. □ 1 minute
30. □ 10 seconds
31. **Which type of hand hygiene method is required in the following situations?**
32. Before palpation of the abdomen □ Rubbing □ Washing □ None
33. Before giving an injection □ Rubbing □ Washing □ None
34. After emptying a bedpan □ Rubbing □ Washing □ None
35. After removing examination gloves □ Rubbing □ Washing □ None
36. After making a patients’ bed □ Rubbing □ Washing □ None
37. After visible exposure to blood □ Rubbing □ Washing □ None
38. **Which of the following should be avoided, as associated with increased likelihood of colonisation of hands with harmful germs?**
39. Wearing jewellery □ Yes □ No
40. Damaged skin □ Yes □ No
41. Artificial fingernails □ Yes □ No
42. Regular use of hand cream □ Yes □ No
43. **In your opinion, what is the average percentage of hospitalised patients who will develop a healthcare-associated infection (between 0 and 100%)?**
    ….% □ I don’t know
44. **In general, what is the impact of a healthcare-associated infection on a patient's clinical outcome?**□ Very low □ Low □ High □ Very high
45. **What is the effectiveness of hand hygiene in preventing healthcare-associated infection?**
    □ Very low □ Low □ High □ Very high
46. **On average, in what percentage of situations requiring hand hygiene do you actually perform hand hygiene, either by handrubbing or handwashing (between 0 and 100%)?**….%
47. **Working according to the hand hygiene guidelines can sometimes be difficult. We would like to know if the following problems interfere with you hand hygiene compliance**

**What would be a reason/reasons for you to not perform hand hygiene on a moment that it is expected?**

1. It takes too much time □      Yes □      No
2. There are not enough facilities □      Yes □      No
3. The hand hygiene procedure makes my skin dry or irritated □      Yes □      No
4. I don't believe hand alcohol is effective in making my hands clean □      Yes □      No
5. Because of my religion I am not allowed to use hand alcohol □      Yes □      No
6. I don't like the smell of hand alcohol □      Yes □      No
   The substance is not convenient (for example too sticky) □      Yes □      No
7. I don't use hand alcohol when my hands are sweaty □      Yes □      No

1. **In your opinion what interventions would improve hand hygiene permanently in your institution? Please write down two interventions.**
   1.

2**.**

*Thank you very much for your time!*

Sebagai petugas kesehatan, anda berinteraksi langsung dengan pasien dalam perawatan sehari-hari, oleh karena itu kami tertarik untuk mengetahui pendapat anda tentang hubungan *healthcare-associated infection* (HCAI) dan *hand hygiene*.

- Pengisian kuesioner ini membutuhkan waktu sekitar 15 menit.
- Setiap pertanyaan hanya boleh memilih **satu jawaban.**
- Silakan baca pertanyaan dengan seksama dan respon secara spontan. Nama dan jawaban anda akan dirahasiakan.
- **Kamus singkat:**
  - **Formulasi *alcohol-based hands-rub*:** preparasi yang mengandung alkohol (cair, jel atau busa) yang didesain untuk membunuh kuman di tangan.
  - **Fasilitas:** pelayanan kesehatan (rumah sakit) tempat survei dilakukan.
  - ***Hands-rubbing*:** mencuci tangan dengan *hands-rub* antiseptik (formulasi berbasis alkohol)**.**
  - ***Hands-washing*:** mencuci tangan dengan air dan sabun antimikroba.

**Tanggal**:

**Jenis kelamin**: □ Perempuan □ Laki-laki

**Profesi**: □ Perawat □ Mahasiswa keperawatan □ Dokter □ Mahasiswa kedokteran □ Lainnya

1. **Manakah dari pernyataan di bawah ini yang merupakan rute utama potensial transmisi silang kuman patogen antar pasien pada fasilitas kesehatan? (hanya satu jawaban)**
2. □ Tangan petugas kesehatan yang tidak bersih
3. □ Sirkulasi udara dalam rumah sakit
4. □ Pasien terpajan permukaan yang terkolonisasi (contoh: tempat tidur, meja, lantai)
5. □ Penggunaan bersama objek non-invasif antar pasien (contoh: stetoskop, pengukur tekanan darah)
6. **Apa yang merupakan sumber kuman tersering penyebab HCAI? (hanya satu jawaban)**
7. □ Sistem perairan di rumah sakit
8. □ Udara rumah sakit
9. □ Kuman memang sudah ada pada pasein
10. □ Lingkungan rumah sakit (permukaan)
11. **Manakah tindakan hand hygiene di bawah ini yang dapat mencegah transmisi kuman ke pasien?**
12. □ Sebelum menyentuh pasien □ Ya □ Tidak
13. □ Segera setelah terpapar cairan tubuh □ Ya □ Tidak
14. □ Segera sebelum melakukan prosedur bersih/aseptik □ Ya □ Tidak
15. □ Setelah terpajan lingkungan sekitar pasien □ Ya □ Tidak
16. **Manakah tindakan hand hygiene di bawah ini untuk mencegah transmisi ke petugas kesehatan?**
17. □ Sebelum menyentuh pasien □ Ya □ Tidak
18. □ Segera setelah terpajan cairan tubuh □ Ya □ Tidak
19. □ Segera sebelum melakukan prosedur bersih/aseptik □ Ya □ Tidak
20. □ Setelah terpapar lingkungan sekitar pasien □ Ya □ Tidak
21. **Manakah pernyataan di bawah ini yang benar mengenai *alcohol-based* *hands-rub* dan cuci tangan dengan sabun dan air (*hands-washing*)?**
22. *Hands- rubbing* lebih cepat untuk membersihkan tangan dibanding *hands-washing* □ Benar □ Salah
23. *Hands-rubbing* lebih menyebabkan kulit kering dibanding *hands-washing* □ Benar □ Salah
24. *Hands-rubbing* lebih efektif melawan kuman dibandingkan *hands-washing* □ Benar □ Salah
25. Hands-washing dan *hands-rubbing* direkomendasikan untuk dilakukan berurutan □ Benar □ Salah
26. **Berapa waktu minimal yang diperlukan untuk *alcohol-based hands-rub* untuk membunuh kuman di tangan?**
27. □ 20 detik
28. □ 3 detik
29. □ 1 menit
30. □ 10 detik
31. **Apa metode *hand hygiene* yang diperlukan untuk situasi berikut ini?**
32. Sebelum palpasi abdomen □ *Rubbing* □ *Washing* □ Tidak perlu
33. Sebelum melakukan injeksi □ *Rubbing* □ *Washing* □ Tidak perlu
34. Setelah mengosongkan *bedpan* □ *Rubbing* □ *Washing* □ Tidak perlu
35. Setelah melepaskan *gloves* yang sudah digunakan □ *Rubbing* □ *Washing* □ Tidak perlu
36. Setelah merapikan tempat tidur pasien □ *Rubbing* □ *Washing* □ Tidak perlu
37. Setelah terpajan oleh darah □ *Rubbing* □ *Washing* □ Tidak perlu
38. **Apa yang harus dihindari dari hal berikut ini dalam kaitannya dengan kolonisasi kuman di tangan?**
39. Menggunakan perhiasan □ Ya □ Tidak
40. Kerusakan kulit □ Ya □ Tidak
41. Kuku buatan □ Ya □ Tidak
42. Penggunaan krim tangan secara teratur □ Ya □ Tidak
43. **Menurut pendapat anda berapa persentase rata-rata pasien yang dirawat di rumah sakit untuk mendapat HCAI (antara 0 dan 100%)?**
    ….% □ Tidak tahu
44. **Secara umum, apa pengaruh HCAI terhadap luaran klinis pasien?**□ Sangat rendah □ Rendah □ Tinggi □ Sangat tinggi
45. **Bagaimana efektifitas *hand hygiene* dalam mencegah HCAI?**
    □ Sangat rendah □ Rendah □ Tinggi □ Sangat tinggi
46. **Dalam situasi yang memerlukan *hand hygiene*, berapa persen rata-rata anda melakukan *hand hygiene*, baik *hands-rubbing* atau *hands-washing* (antara 0 dan 100%)?**….%
47. **Bekerja sesuai dengan panduan *hand hygiene* kadang sulit untuk dilakukan. Kami ingin mengetahui masalah yang anda hadapi terkait kepatuhan cuci tangan. Apakah alasan anda sehingga tidak melakukan cuci tangan (jawaban boleh lebih dari satu)?**
48. Waktu yang diperlukan terlalu banyak □      Ya □      Tidak
49. Fasilitas tidak tersedia □      Ya □      Tidak
50. Prosedur *hand hygiene* membuat kulit kering dan iritasi □      Ya □      Tidak
51. Saya tidak yakin bahwa hands-rubbing dengan alkohol tidak efektif untuk membersihkan tangan □      Ya □      Tidak
52. Karena kepercayaan (agama) saya, saya tidak dibolehkan menggunakan alkohol

□      Ya □      Tidak

1. Saya tidak suka bau alkohol. Substansinya tidak nyaman (terlalu lengket) □      Ya □      Tidak
2. Saya tidak menggunakan alcohol-based hands-rub jika tangan saya dalam keadaan berkeringat □      Ya □      Tidak

1. **Tuliskan pendapat anda intervensi apa yang harus dilakukan untuk memperbaiki praktik cuci tangan di institusi anda? Tuliskan 2 interveni.**
   1.

2**.**

*Terima kasih atas waktu anda!*
