# Supplementary material for: A multifaceted hand hygiene improvement program on the intensive care units of the National Referral Hospital of Indonesia in Jakarta
Source: Antimicrob Resist Infect Control. 2019 Jun 3;8:93. doi: 10.1186/s13756-019-0540-4 (PMC6547605; doi:10.1186/s13756-019-0540-4)
Supplement: Supplementary file 2 — Table S1. Survey results regarding hand hygiene knowledge questions. (DOCX 17 kb) [file 13756_2019_540_MOESM2_ESM.docx]

**Additional file 2.**

**Table.** Survey results regarding hand hygiene knowledge questions.

|  | Baseline (%)  correct answer | Post-intervention (%) correct answer | P-value  (two-sided) |
| --- | --- | --- | --- |
| **Number of correct answers per HCW (median)** | 15 (60) | 22(88) | **<0.001** |
| Question 1 | 79 (81.4) | 50 (69.4) | 0.099 |
| Question 2 | 4 (4.1) | 54 (75.0) | <0.001 |
| Question 3a | 92 (94.8) | 72 (100) | 0.072 |
| Question 3b | 17 (17.5) | 50 (69.4) | <0.001 |
| Question 3c | 83 (85.6) | 69 (95.8) | 0.037 |
| Question 3d | 17 (17.5) | 49 (68.1) | <0.001 |
| Question 4a | 23 (23.7) | 55 (76.4) | <0.001 |
| Question 4b | 86 (88.7) | 67 (93.1) | 0.430 |
| Question 4c | 20 (20.6) | 56 (77.8) | <0.001 |
| Question 4d | 83 (85.7) | 66 (91.7) | 0.336 |
| Question 5a | 74 (76.3) | 69 (95.8) | <0.001 |
| Question 5b | 15 (15.5) | 50 (69.4) | <0.001 |
| Question 5c | 14 (14.4) | 38 (52.8) | <0.001 |
| Question 5d | 26 (26.8) | 45 (62.5) | <0.001 |
| Question 6 | 69 (71.1) | 69 (95.8) | <0.001 |
| Question 7a | 80 (82.5) | 70 (97.2) | 0.003 |
| Question 7b | 69 (71.1) | 69 (95.8) | <0.001 |
| Question 7c | 90 (92.8) | 72 (100.0) | 0.021 |
| Question 7d | 29 (29.9) | 48 (66.7) | <0.001 |
| Question 7e | 45 (46.4) | 64 (88.9) | <0.001 |
| Question 7f | 89 (91.8) | 71 (98.6) | 0.080 |
| Question 8a | 84 (86.6) | 70 (97.2) | 0.026 |
| Question 8b | 61 (62.9) | 43 (59.7) | 0.750 |
| Question 8c | 78 (80.4) | 65 (90.3) | 0.088 |
| Question 8d | 58 (59.8) | 63 (87.5) | <0.001 |

Differences between baseline and post-intervention data were assessed using Fisher’s exact test (for individual questions) and Mann-Whitney test (for the total number of correct answers per HCW). The written text of each question can be retrieved from Additional file 1.
